# K_v_1.3 channel blockade with the Vm24 scorpion toxin attenuates the CD4^+^ effector memory T cell response to TCR stimulation

**DOI:** 10.1186/s12964-018-0257-7

**Published:** 2018-08-14

**Authors:** José Ignacio Veytia-Bucheli, Juana María Jiménez-Vargas, Erika Isabel Melchy-Pérez, Monserrat Alba Sandoval-Hernández, Lourival Domingos Possani, Yvonne Rosenstein

**Affiliations:** 1 Departamento de Medicina Molecular y Bioprocesos, Instituto de Biotecnología, Universidad Nacional Autónoma de México, Av. Universidad 2001, Col. Chamilpa, 62210 Cuernavaca, Morelos Mexico; 2 Posgrado en Ciencias Bioquímicas, Universidad Nacional Autónoma de México, Mexico City, Mexico

**Keywords:** K_v_1.3 potassium channel, Vm24 toxin, Effector memory T cells, Proteomics, Autoimmune disease

## Abstract

**Background:**

In T cells, the K_v_1.3 and the K_Ca_3.1 potassium channels regulate the membrane potential and calcium homeostasis. Notably, during T_EM_ cell activation, the number of K_v_1.3 channels on the cell membrane dramatically increases. K_v_1.3 blockade results in inhibition of Ca^2+^ signaling in T_EM_ cells, thus eliciting an immunomodulatory effect. Among the naturally occurring peptides, the Vm24 toxin from the Mexican scorpion *Vaejovis mexicanus* is the most potent and selective K_v_1.3 channel blocker known, which makes it a promissory candidate for its use in the clinic. We have shown that addition of Vm24 to TCR-activated human T cells inhibits CD25 expression, cell proliferation and reduces delayed-type hypersensitivity reactions in a chronic inflammation model. Here, we used the Vm24 toxin as a tool to investigate the molecular events that follow K_v_1.3 blockade specifically on human CD4^+^ T_EM_ cells as they are actively involved in inflammation and are key mediators of autoimmune diseases.

**Methods:**

We combined cell viability, activation, and multiplex cytokine assays with a proteomic analysis to identify the biological processes affected by K_v_1.3 blockade on healthy donors CD4^+^ T_EM_ cells, following TCR activation in the presence or absence of the Vm24 toxin.

**Results:**

The peptide completely blocked K_v_1.3 channels currents without impairing T_EM_ cell viability, and in response to TCR stimulation, it inhibited the expression of the activation markers CD25 and CD40L (but not that of CD69), as well as the secretion of the pro-inflammatory cytokines IFN-γ and TNF and the anti-inflammatory cytokines IL-4, IL-5, IL-9, IL-10, and IL-13. These results, in combination with data from the proteomic analysis, indicate that the biological processes most affected by the blockade of K_v_1.3 channels in a T cell activation context were cytokine-cytokine receptor interaction, mRNA processing via spliceosome, response to unfolded proteins and intracellular vesicle transport, targeting the cell protein synthesis machinery.

**Conclusions:**

The Vm24 toxin, a highly specific inhibitor of K_v_1.3 channels allowed us to define downstream functions of the K_v_1.3 channels in human CD4^+^ T_EM_ lymphocytes. Blocking K_v_1.3 channels profoundly affects the mRNA synthesis machinery, the unfolded protein response and the intracellular vesicle transport, impairing the synthesis and secretion of cytokines in response to TCR engagement, underscoring the role of K_v_1.3 channels in regulating T_EM_ lymphocyte function.

**Electronic supplementary material:**

The online version of this article (10.1186/s12964-018-0257-7) contains supplementary material, which is available to authorized users.

## Background

Ion transport through ion channels is essential to regulate the membrane potential, the signaling by calcium (Ca^2+^), magnesium, zinc and other divalent cations, as well as downstream events such as gene expression, apoptosis, proliferation, development, and migration [1, 2]. Immune cells express a variety of ion channels and transporters that allow the flux of ions across the plasma membrane and the membrane of intracellular organelles. In T cells, the interaction of the T cell receptor (TCR) with its cognate antigen leads to an increase in the intracellular Ca^2+^ concentration, regulating numerous downstream signaling pathways that control clonal expansion, differentiation and cytokine production [1]. Following intracellular Ca^2+^ stores depletion, the electrochemical potential required for Ca^2+^ entry through Ca^2+^ release-activated Ca^2+^ channels (CRAC) in the plasma membrane is regulated by the efflux of potassium cations to the extracellular space, a process controlled by potassium channels [3]. In T lymphocytes, the voltage-gated potassium channel K_v_1.3 and the calcium-activated potassium channel K_Ca_3.1 regulate the membrane potential and calcium homeostasis [4] by operating at different levels of the Ca^2+^ signaling pathway. Furthermore, the K_v_1.3 and the K_Ca_3.1 potassium channels cluster with the CRAC calcium channel at the immunological synapse and regulate its function [3, 4]. The K_v_1.3 channels four identical subunits contain a voltage sensor and are activated by membrane depolarization [5]. Notably, upon activation, the number of K_v_1.3 channels of effector memory T (T_EM_) cells dramatically increases, while that of K_Ca_3.1 channels remains constant, underscoring a role of K_v_1.3 channels in the decision making process of T_EM_ lymphocytes [6]. T_EM_ lymphocytes rapidly and copiously produce and release inflammatory and cytotoxic mediators such as IFN-γ, IL-4, and perforin. They lack CCR7 and CD62L, two receptors involved in homing to the lymph nodes, but the expression of the receptors for inflammatory cytokines CCR1, CCR3 and, CCR5 allows them to recirculate between the blood and inflammatory foci [7–11]. Blocking K_v_1.3 channels in T_EM_ cells has been reported to reduce the influx of Ca^2+^, resulting in a T_EM_-specific immunomodulatory effect [4, 12, 13], without compromising naïve and central memory T (T_CM_) lymphocytes’ effector functions, such as protection against pathogens, and T and B cells crosstalk for the generation of high affinity protective antibodies and isotype switching [14, 15]. Autoreactive cells found in multiple sclerosis, rheumatoid arthritis and type I diabetes mellitus lesions exhibit a T_EM_ phenotype and are key mediators in the pathogenesis of these autoimmune diseases [6, 16], stressing the need for restraining these cells.

Potent K_v_1.3 channel blockers have been found in animal venoms. These molecules interact with the channel though a pharmacophore called “functional dyad”, consisting of a blocking lysine and an aromatic residue located around 7 Å apart. The lysine interacts with acidic residues on the channel selectivity filter, blocking the conduction pore, and preventing the passage of ions through it [17]. Several scorpion toxins (margatoxin, noxiustoxin, kaliotoxin, charybdotoxin, agitoxin-2, OSK1, hongotoxin, anuroctoxin), anemone toxins (ShK) and even peptides from parasitic worms (AcK1, BmK1) have been shown to block the K_v_1.3 potassium channel with pico- or nanomolar affinities. Particularly, the anemone ShK peptide has been reported to suppress the T_EM_ cell proliferation and pro-inflammatory cytokine secretion without affecting naïve or T_CM_ lymphocytes [6, 16, 18]. Unfortunately, these toxins are promiscuous and affect other related potassium channels (K_v_1.1, K_v_1.2, K_v_1.6, K_v_1.7) necessary for the activity of neurons and muscle cells, eventually causing severe adverse effects and even death [17, 19–24].

Among the naturally occurring peptides, the 36 amino acid toxin Vm24, isolated from the Mexican scorpion *Vaejovis mexicanus*, is the most potent (K_d_ = 2.9 pM) and selective (> 1500-fold affinity over other assayed potassium channels) K_v_1.3 channel blocker known, what makes it a very promissory candidate for its use in the clinic [23]. This peptide, similar to other scorpion ion channel modulating toxins, has a cysteine-stabilized α/β structural motif, formed by a short α-helix joined by four disulfide bridges to a triple-stranded antiparallel β-sheet. This structural motif, in combination with the C-terminal amidation, confers stability to the toxin [25]. We have shown that addition of Vm24 to TCR-activated human T cells inhibits calcium-mediated cell signaling and generates a dose-dependent inhibition of CD25 expression and cell proliferation. Furthermore, it reduces delayed-type hypersensitivity reactions in rats in a chronic inflammation model [23]. To achieve a deeper understanding of the role of K_v_1.3 ion channels in the immune response, to identify the biological processes affected by K_v_1.3 blockade, and to better characterize the potential pharmacological use of the Vm24 peptide, we evaluated the cytokine secretion and proteomic profiles of CD4^+^ T_EM_ cells isolated from healthy donors following TCR activation, in the presence or absence of the Vm24 toxin.

## Methods

### Vm24 and ShK toxins

The Vm24 peptide, the generous gift of Dr. Georgina Gurrola-Briones, was prepared by chemical synthesis according to previously published work [25]. The ShK toxin was purchased from Alomone Labs (Jerusalem, Israel).

### CD4^+^ T_EM_ lymphocytes purification

This procedure was approved by the Bioethics Committee of the Instituto de Biotecnología. Buffy coats from anonymized healthy donors were obtained from the *Centro Estatal de la Transfusión Sanguínea* (Cuernavaca, Morelos, Mexico). Mononuclear cells were separated through Ficoll-Paque PLUS (GE Healthcare Bio-Sciences AB, Uppsala, Sweden) density gradient centrifugation. Cells obtained were resuspended in RPMI-1640 medium (HyClone, GE Healthcare Life Sciences, Logan, UT, USA) supplemented with 10% fetal calf serum (By Productos, Guadalajara, Jalisco, Mexico) and incubated in 100 mm tissue-culture treated polystyrene dishes (8 × 10^7^ cells/dish) at 37 °C in 5% CO_2_ overnight. Non-adherent cells were recovered in arrest medium (RPMI-1640 medium supplemented with 2% fetal calf serum), and incubated in the same medium at 37 °C in 5% CO_2_ for 24 h. CD4^+^ T_EM_ lymphocytes were purified by magnetic cell sorting (negative selection) with the CD4^+^ Effector Memory T Cell Isolation Kit (Miltenyi Biotec GmbH, Bergisch Gladbach, Germany). Briefly, non-CD4^+^ T_EM_ cells were labeled with a monoclonal antibody cocktail (biotin-conjugated anti-CD8, CD14, CD15, CD16, CD19, CD34, CD36, CD45RA, CD56, CD123, CD235a, TCRγ/δ and APC-conjugated anti-CCR7). Subsequently, the preparation was incubated with anti-biotin and anti-APC secondary antibodies conjugated with magnetic MicroBeads. The cell suspension was transferred to an LD Column (Miltenyi Biotec GmbH) placed on a MidiMACS Separator (Miltenyi Biotec GmbH) permanent magnet. The CD4^+^ T_EM_ lymphocytes were recovered by elution, and purity (CD3, CD4, CD45RO and CCR7 expression) was determined by flow cytometry.

### Electrophysiological studies

Blockade of K_v_1.3 potassium channels by the Vm24 toxin was evaluated on purified CD4^+^ T_EM_ lymphocytes. Whole-cell currents were measured in voltage-clamped cells using a MultiClamp 700B (Molecular Devices, LLC, Sunnyvale, CA, USA) amplifier connected to a computer with Digidata 1440A (Molecular Devices, LLC) digitizer hardware. For data analysis, the pCLAMP 10 (Molecular Devices, LLC) software package was used. Cells were observed with an Eclipse TS100 (Nikon Instruments Inc., Melville, NY, USA) inverted microscope. Pipettes were pulled from G120 T-4 borosilicate glass capillaries (Warner Instruments, LLC, Hamden, CT, USA) in two stages, which resulted in electrodes with 3 to 5 MΩ resistance in the bath. The bath solution consisted of 145 mM NaCl, 5 mM KCl, 1 mM MgCl_2_, 2.5 mM CaCl_2_, 5.5 mM glucose and 10 mM HEPES (pH 7.35). The pipette filling solution contained 140 mM KF, 2 mM MgCl_2_, 1 mM CaCl_2_, 10 mM HEPES and 11 mM EGTA (pH 7.22). For currents measurements from K_v_1.3 channels, a depolarizing pulse to + 50 mV was applied for 14 milliseconds from a holding potential of − 120 mV. The protocol was repeated every 15 s. The Vm24 toxin was perfused to the cells at a concentration of 1 nM.

### CD4^+^ T_EM_ lymphocytes stimulation

CD4^+^ T_EM_ lymphocytes were divided in five groups: a) unstimulated cells, b) unstimulated cells + Vm24 (1 nM), c) anti-CD3 stimulated cells, d) anti-CD3 stimulated cells + Vm24 (1 nM), and e) anti-CD3 stimulated cells + ShK (1 nM). For TCR-specific stimulation, anti-human CD3e (clone OKT3, home purified) monoclonal antibody was bound to the surface of 24-well polystyrene cell culture plates at 2 μg/cm^2^, for two hours at 37 °C in phosphate-buffered saline (PBS). Wells were washed three times with PBS to remove the unbound antibody and 1 mL of the cell suspension (1 × 10^6^ cells/mL) per well was plated. When indicated, cells were incubated with the Vm24 or ShK toxins (1 nM) five minutes before the onset of stimulation. Plates were incubated at 37 °C in 5% CO_2_ for the indicated times. As experiments were performed with cells from different donors, the expression of the activation marker CD25 (evaluated by flow cytometry) of cells stimulated in the presence or absence of Vm24, was used as internal quality control for all samples.

### Cytokine secretion profile

The supernatants from CD4^+^ T_EM_ lymphocytes activated as indicated above were collected, and the secretion of cytokines was evaluated with the LEGENDplex Human Th Cytokine Panel 13-plex (BioLegend, San Diego, CA, USA) and flow cytometry, following the manufacturer’s instructions. Data were collected on a BD FACSCanto II (BD Biosciences, San Jose, CA, USA) flow cytometer with the BD FACSDiva (version 6.1.3, BD Biosciences) software, and analyzed with the LEGENDplex Data Analysis Software (BioLegend).

### Quantitative proteomic analysis

The quantitative proteomic analysis was performed on CD4^+^ T_EM_ lymphocytes from three independent donors. Cells from each donor were divided in four conditions (2 × 10^6^ cells/condition): a) unstimulated cells, b) unstimulated cells + Vm24 (1 nM), c) anti-CD3 stimulated cells, and d) anti-CD3 stimulated cells + Vm24 (1 nM), yielding a total of 12 samples. After a 24 h incubation period, cells were washed with PBS and disrupted in 200 μL of lysis solution (2 M urea, 7 M thiourea, 4% CHAPS, 50 mM DTT) supplemented with cOmplete (Roche Diagnostics GmbH, Mannheim, Germany) protease inhibitor cocktail and PhosSTOP (Roche Diagnostics GmbH) phosphatase inhibitor cocktail. The preparations were incubated on ice for 20 min and centrifuged at 17,000 x *g* at 4 °C for 5 min to remove insoluble material. Total protein in the supernatant was quantified with the 2-D Quant Kit (GE Healthcare Bio-Sciences Corp, Piscataway, NJ, USA), and 30 μg of protein were taken from each sample and brought to 250 μL with 10× TE buffer (100 mM Tris-HCl, 10 mM EDTA, pH 8.0), 100 μL of 0.3% sodium deoxycholate were added and protein was sequentially precipitated with 72% trichloroacetic acid, followed by 90% acetone as previously reported [26, 27]. Samples were vacuum-dried and sent to the Proteomics Facility of the *Institut de Recherches Cliniques* (Montreal, Canada). Proteins were reduced with DTT, alkylated with iodoacetamide and digested with trypsin, and the resulting peptides were analyzed by nano-liquid chromatography coupled to tandem mass spectrometry (MS/MS) as previously reported [26, 27]. The Scaffold (version 4.4.7, Proteome Software, Inc., Portland, OR, USA) software was used to validate MS/MS-based peptide and protein identifications. Peptide identifications were accepted if they exceeded the specific database search engine thresholds, calculated as -10log (*p*), where *p* is the probability that the observed match between the experimental data and the database sequence is a random event. Mascot (Matrix Science Inc., Boston, MA, USA) identification requires that ion scores be at least greater than the associated identity scores and 20, 15 and 15 for peptides with double, triple, and quadruple charges, respectively. X! Tandem (The Global Proteome Machine, [28]) identifications required at least –log (expected) scores greater than 2.0. Peptide identifications were accepted if they could be established at greater than 95% probability as specified by the PeptideProphet algorithm [29]. Protein identifications were accepted if they could be established at greater than 99% probability [30]. Proteins that contained similar peptides and could not be differentiated based on MS/MS analysis alone were grouped to satisfy the principles of parsimony. For protein quantitation, the exponentially modified protein abundance index (emPAI) value was used [31].

### Interaction networks

For protein physical/functional interaction networks identification and functional enrichments specific for Biological Process (GO) and KEGG Pathways, the STRING database (version 10.5, [32]) was used. For interaction networks, proteins were linked based on neighborhood, gene fusion, co-expression, co-occurrence, experimental evidences, existing databases and text-mining criteria with a minimum required interaction score of 0.7 (high confidence).

### Flow cytometry

Cell viability was assessed with the Fixable Viability Dye eFluor 780 (Life Technologies, Carlsbad, California, USA). Cells were incubated with the dye (500 μL of a 1:100,000 dilution in PBS) at 4 °C for 30 min in the dark, before analysis by flow cytometry. Changes in forward scatter (FSC) and positive staining with the viability dye were considered as markers of cell death. For fluorescent antibody staining, cells were washed with FACS solution (PBS supplemented with 0.5% bovine serum albumin and 0.1% sodium azide). Fc receptors were blocked with 100 μL of 10% human serum at 4 °C for 30 min, and cells were stained in a 150 μL final volume with the PE anti-human CD3d (clone 7D6, Caltag Laboratories, Burlingame, CA, USA), PerCP anti-human CD3e (clone SK7, BioLegend), TC anti-human CD4 (clone S3.5, Invitrogen Camarillo, CA, USA), PE anti-human CD45RO (clone UCHL1, BioLegend), APC anti-human CCR7 (clone G043H7, BioLegend), APC anti-human CD25 (clone BC96, BioLegend), PE anti-human CD69 (clone FN50, BioLegend), and APC anti-human CD40L (clone 24–31, eBioscience, San Diego, CA, USA) fluorescent antibodies where indicated at 4 °C for 30 min and washed. For IRF4 and Hsp90 detection, cells were fixed with 2% paraformaldehyde in PBS at 37 °C for 10 min, washed and incubated on ice with 500 μL of permeabilization buffer (0.5% Triton X-100, 0.2 μg/mL EDTA and 1% bovine serum albumin in PBS) for 15 min. After removal of the detergent solution, cells were further permeabilized with 90% methanol for an additional hour at − 20 °C and stained with PE anti-IRF4 (clone IRF4.3E4, BioLegend) or anti-Hsp90α/β (clone 3H3C27, BioLegend) antibodies, followed by Alexa Fluor 647 goat anti-mouse IgG (Invitrogen, Eugene, OR, USA) second step. Samples were acquired on a BD FACSCanto II (BD Biosciences) flow cytometer with the BD FACSDiva (BD Biosciences) software and analyzed using the FlowJo (version 8.7, FlowJo, LLC, Ashland, OR, USA) software.

### Statistical analysis

Data analysis and graphics creation were performed with the OriginPro 7 (OriginLab Corporation, Northampton, MA, USA) software. The heat map was generated using the open-source software MultiExperiment Viewer (version 4.9.0, [33]). For statistical significance, one-way ANOVA test (*p* < 0.05) was used as a first threshold for differences across treatment groups, followed by a Fisher’s Least Significant Difference (LSD) post-hoc test (*p* < 0.05).

## Results

### K_v_1.3 channel blockade by the Vm24 toxin does not compromise cell viability but decreases the activation of T_EM_ cells

K_v_1.3 is the dominating potassium channel in T_EM_ cells, and a potential target to treat chronic inflammation by selectively compromising T_EM_ cells without undermining the function of naïve and T_CM_ cells that rather depend on the K_Ca_3.1 channels [6]. We previously reported that by specifically blocking the K_v_1.3 channel, the Vm24 toxin inhibited T cell activation and proliferation [8]. As these results were obtained with total T cells, here we evaluated the capacity of the Vm24 peptide to specifically inhibit the CD4^+^ T_EM_ cells function. As a control for K_v_1.3 channel blockade, we used the ShK toxin from the Caribbean Sea anemone *Stichodactyla helianthus*, a recognized K_v_1.3 channels inhibitor [21].

**Fig. 1:**
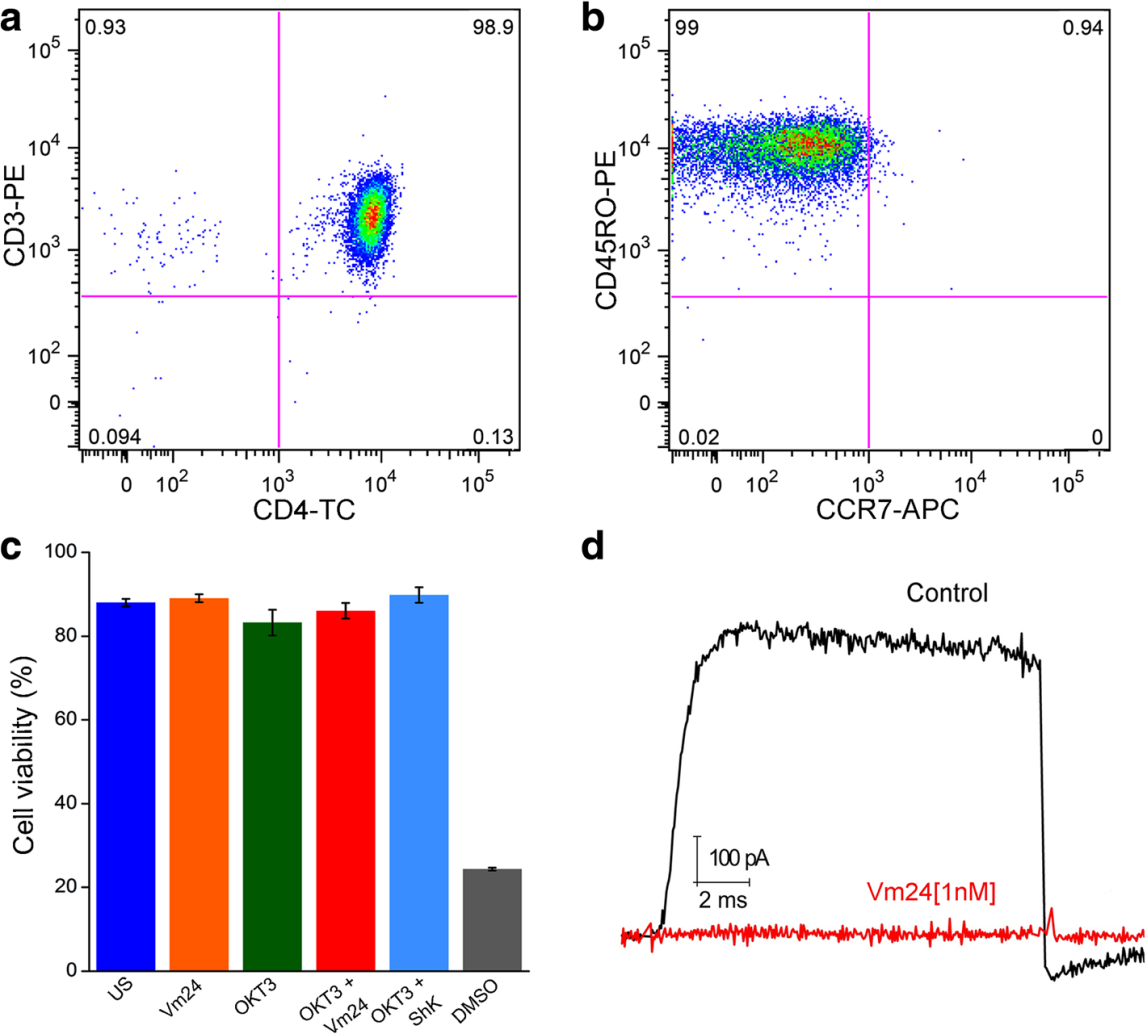
K_v_1.3 channel blockade does not compromise cell viability. Purified CD4^+^ T_EM_ cells were stained for the (**a**) CD3, CD4 and (**b**) CD45RO and CCR7 surface markers. (**c**) Cell viability was assessed following a 24 h culture period with the Fixable Viability Dye eFluor 780. Changes in FSC and positive staining with the viability dye were considered as cell death markers. For death positive control, 30% dimethyl sulfoxide (DMSO) was added to the cells for 30 min. Data from three independent experiments are shown as mean ± SEM (standard error of mean). (**d**) K_v_1.3 channels currents were measured by patch clamp in whole cell mode. Currents were evoked by a depolarizing pulse to + 50 mV from a − 120 mV holding potential. The Vm24 toxin was perfused to the cells at a concentration of 1 nM

CD4^+^ T_EM_ cells isolated from peripheral blood of healthy donors were positive (> 95%) for the CD3, CD4 and CD45RO surface markers and negative for the CCR7 chemokine receptor (Fig. 1a, b). These markers were verified before and after activation, and under all conditions, the terminally differentiated CD4^+^ T_EM_ cells maintained their phenotype (CD45RO^+^ and CCR7^−^), as previously reported [34, 35]. Since the main goal of this study was to perform a functional analysis of TCR-activated T_EM_ cells in the presence of the Vm24 peptide, we first evaluated whether blocking K_v_1.3 compromised cell viability.

Following a 24, 48 and 96 h culture period in the presence of the Vm24 peptide or the ShK toxin, CD4^+^ T_EM_ cells viability was not impaired either in quiescent or OKT3-activated cells. As death positive control, 30% dimethyl sulfoxide (DMSO) was added to the cells for 30 min (Fig. 1c and Additional file 1a, b).

The ability of the synthetic Vm24 toxin to block CD4^+^ T_EM_ cells K_v_1.3 channels was measured by patch clamp in whole cell mode. Consistent with our previous report [23], the Vm24 toxin (1 nM) completely inhibited the current of K_v_1.3 channels in CD4^+^ T_EM_ lymphocytes. Moreover, the K_v_1.3 channel current was not recovered after a 10 min wash-out period, indicative of the slow dissociation rate of the channel-toxin complex (Fig. 1d). Although concentrations below 100 pM of the Vm24 toxin completely block K_v_1.3 currents [23], a 1 nM concentration was used to ensure complete blockade of the channels throughout the entire culture time. Considering that the estimated K_d_ for K_Ca_3.1 channels (also present in T cells) is at least 4500-fold higher than that for the K_v_1.3 channels [23], it is expected that the remaining current on K_Ca_3.1 channels is still 95% in the presence of 1 nM Vm24, strongly indicating that the effects observed on the T_EM_ cells result exclusively of K_v_1.3 channel blockade.

**Fig. 2:**
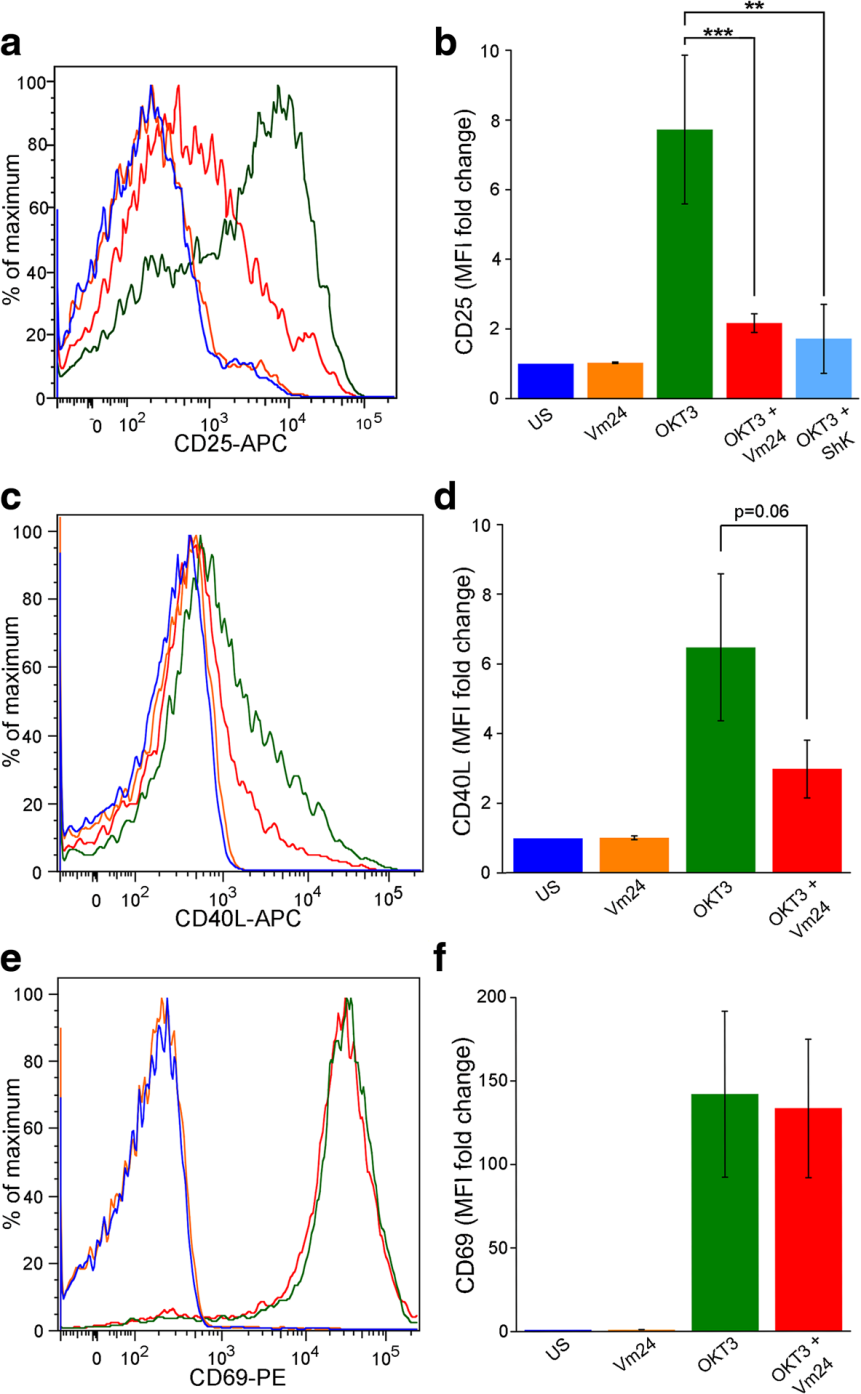
K_v_1.3 channel blockade decreases the expression of CD25 and CD40L, but not that of CD69. CD4^+^ T_EM_ cells were stimulated through the TCR with plate-bound OKT3 in the presence or absence of Vm24 or ShK (1 nM) toxins. After 24 h of culture, cells were stained for (**a**) CD25, (**c**) CD40L and (**e**) CD69. The histogram of a representative donor for each marker is shown. (**b**, **d**, **f**) Data from 3 to 6 independent experiments are shown as mean ± SEM. The color coding for histograms and bars is maintained. Significance of pairwise comparisons between groups is indicated with stars (*p < 0.05, **p < 0.01, ***p < 0.001)

Finally, we evaluated the effect of blocking K_v_1.3 channels on the expression of the early activation markers CD25 (*IL2RA*), CD40L (*CD40LG*) and CD69 (*CD69*) in response to TCR ligation for 24 h. The Vm24 toxin significantly prevented the TCR-mediated CD25 upregulation, similar to the ShK toxin (Fig. 2a, b). Vm24 also prevented the upregulation of CD40L in all subjects (Fig. 2c, d), although this was not statistically significant (*p* = 0.06). Contrary to what we expected, CD69 upregulation was not affected by Vm24 (Fig. 2e, f), suggesting that the K_v_1.3 channels do not participate in the cytoplasm to the plasma membrane translocation of this molecule. Altogether these data indicate that the Vm24 toxin did not compromise cell viability, though it effectively blocked the K_v_1.3 channels on T_EM_ cells, resulting in diminished activation.

### K_v_1.3 channel blockade by the Vm24 toxin decreases the production of pro- and anti-inflammatory cytokines

**Fig. 3:**
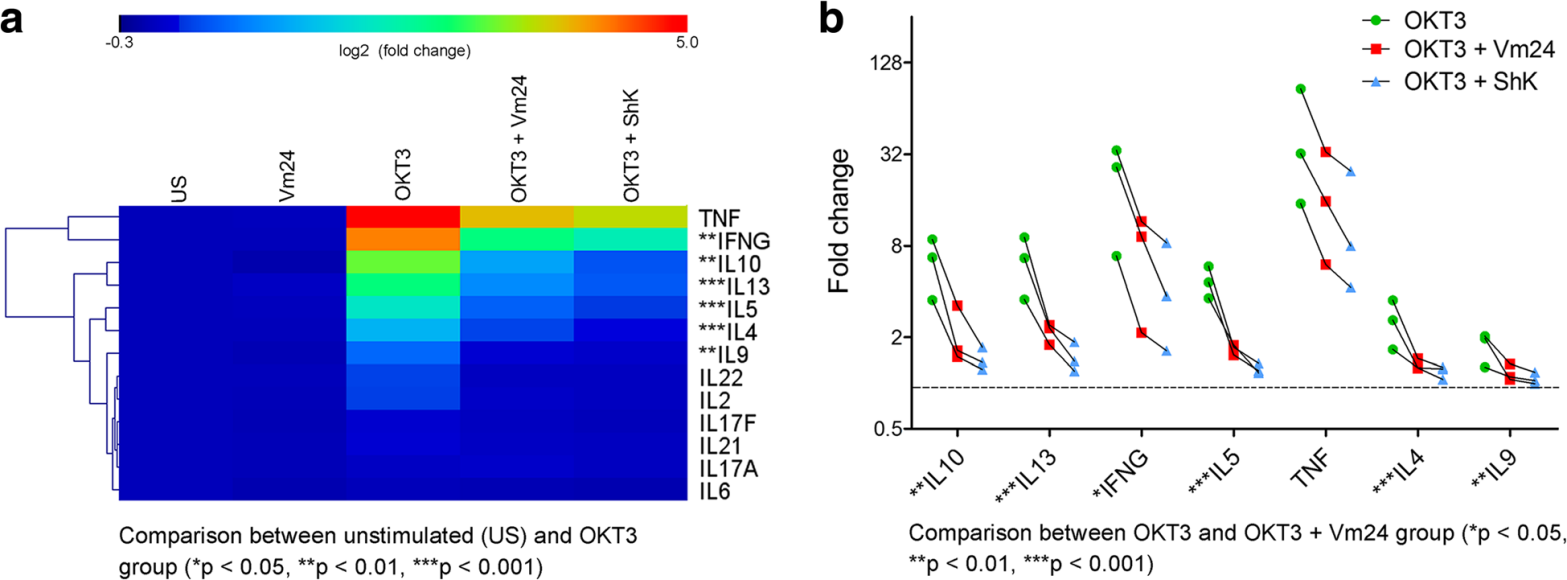
K_v_1.3 channel blockade decreases the production of pro- and anti-inflammatory cytokines. (**a**) Heat map representation of cytokines levels in CD4^+^ T_EM_ cells supernatants. Cells were stimulated for 24 h with plate-bound OKT3 in the presence or absence of Vm24 or ShK (1 nM) toxins and supernatants were collected and analyzed with a multiplex assay. All conditions were normalized to the unstimulated (US) control, and the relative abundance (fold change) of each cytokine is indicated by a gradient of color from blue (low abundance) to red (high abundance). The heat map was generated with the data from three independent donors using the Manhattan distance metric and hierarchical clustering based on average linkage. To identify cytokines modified upon TCR engagement, pairwise comparison between the unstimulated and the OKT3-treated group was performed. Proteins that show at least 1.5-fold change and significant difference (*p* < 0.05) are identified in the heat map with stars (*p < 0.05, **p < 0.01, ***p < 0.001). (**b**) Cytokines that showed increased levels following OKT3 stimulation (Fig. 3a) are plotted. Fold change of OKT3, OKT3 + Vm24 and OKT3 + ShK groups normalized to the unstimulated control are shown. The dotted line indicates the protein levels in the supernatants of unstimulated cells. Data from three independent donors are plotted, and significance of pairwise comparisons between the OKT3 and the OKT3 + Vm24 group are indicated with stars (*p < 0.05, **p < 0.01, ***p < 0.001)

To characterize the impact of blocking the K_v_1.3 channel on TCR activated CD4^+^ T_EM_ lymphocytes function, we analyzed their cytokine secretion profile in the presence of the Vm24 and ShK peptides. Cells were left unstimulated or were activated for 24 h with plate-bound OKT3 in the presence or absence of the toxins, and the presence of cytokines in the supernatant was evaluated with a multiplex assay. Cytokines that showed at least 1.5-fold change and statistically significant differences (*p* < 0.05), were identified. Consistent with previous reports [7, 8, 36], stimulating purified CD4^+^ T_EM_ cells through the TCR induced the secretion of high quantities of the pro-inflammatory cytokine IFN-γ (*IFNG*) as well as that of the anti-inflammatory cytokines IL-4 (*IL4*), IL-5 (*IL5*), IL-9 (*IL9*), IL-10 (*IL10*) and IL-13 (*IL13*), but reduced levels of IL-2 (*IL2*). The level of the pro-inflammatory cytokine TNF (*TNF*) was also markedly increased in all samples, yet not in a statistically significant manner, probably reflecting variability between individuals (Fig. 3a). Although CD4^+^ T_EM_ cells are the principal IL-17-producing population [37], under our experimental conditions (24 h post-OKT3 stimulation), we detected very low levels of IL-17A (*IL17A*) and IL-17F (*IL17F*), likely resulting of the fact that this is a family of late expression cytokines, with secretion peaking around day 6 after activation (Fig. 3a). Pairwise comparison between the OKT3 and the OKT3 + Vm24-treated group showed that activating the cells in the presence of the Vm24 toxin resulted in significantly lower levels in the secretion of IFN-γ, IL-4, IL-5, IL-9, IL-10 and IL-13. Vm24 also reduced the TNF increase by at least 50% in all subjects, although this was not statistically significant, probably due also to inter-individual variation (Fig. 3b). Similar to Vm24, the ShK toxin lowered cytokine production resulting of TCR-mediated activation of CD4^+^ T_EM_ cells.

### K_v_1.3 channel blockade targets the protein synthesis machinery

**Fig. 4:**
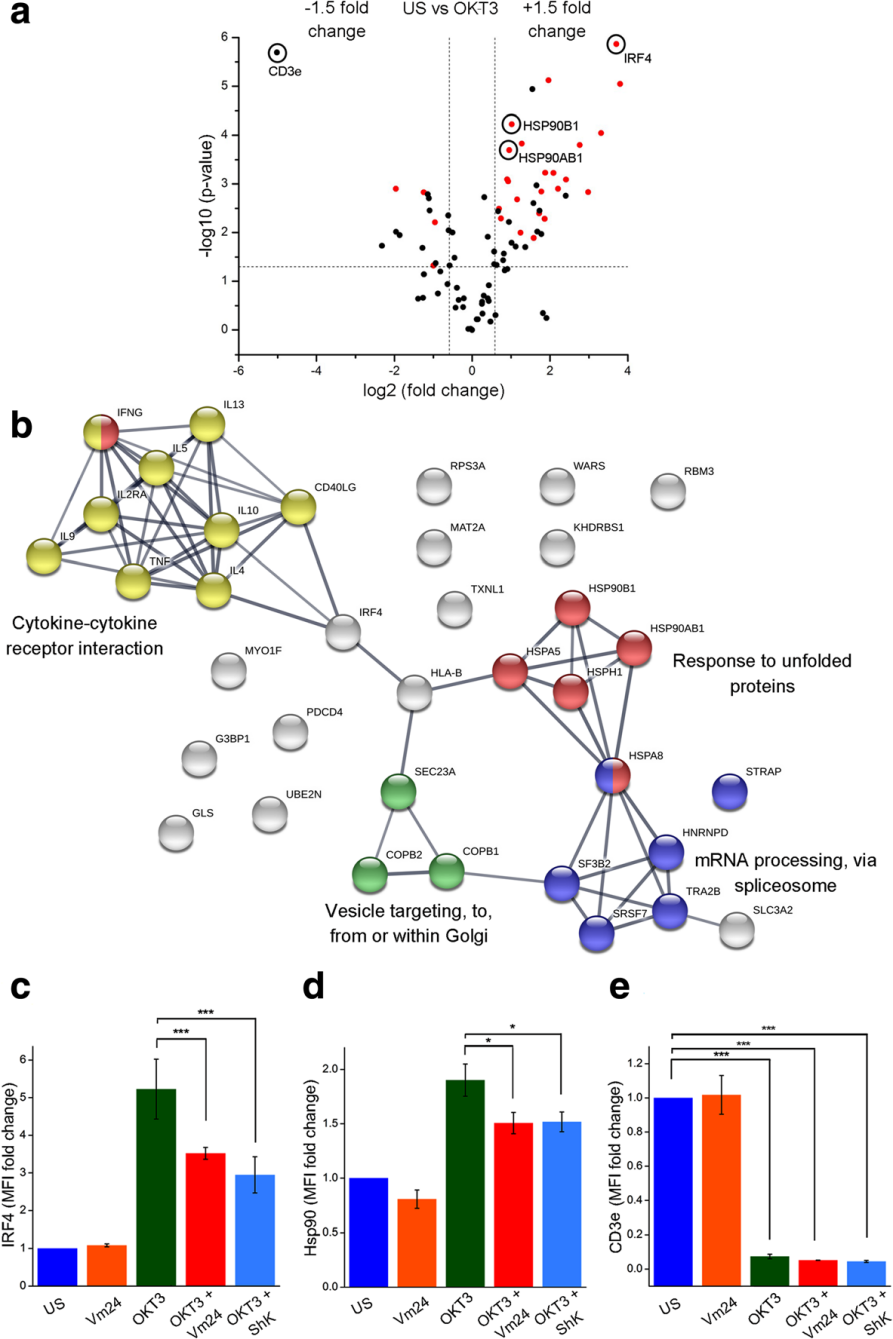
K_v_1.3 channel blockade modifies the proteome, targeting the protein synthesis machinery. (**a**) Mass spectrometry-based quantitative proteomic analysis on CD4^+^ T_EM_ cells. Cells from three independent donors were stimulated for 24 h with plate-bound OKT3 in the presence or absence of Vm24 (1 nM) and total proteins were analyzed by nano-liquid chromatography coupled to MS/MS. All conditions were normalized to the unstimulated (US) control. Pairwise comparison between the unstimulated and the OKT3-treated group was performed to identify proteins modified upon TCR engagement. Proteins that show at least 1.5-fold change in either direction, and significant difference (*p* < 0.05) are identified in the upper right and left segments of the volcano plot. Within the OKT3-regulated proteins, a pairwise comparison (*p* < 0.05) between the OKT3 and the OKT3 + Vm24-treated group was performed to identify proteins (red spots) affected by K_v_1.3 channel blockade, in a T cell activation context. Note that these 27 proteins preserved the same trend of change than when cells where stimulated with OKT3 only, yet the amplitude of the change was reduced. (**b**) Protein–protein interaction network from significantly (*p* < 0.05) modified proteins affected by K_v_1.3 channel blockade in a T cell activation context. The combination of the proteomic data set with the activation markers and the cytokine profile was used to generate a protein physical/functional interaction network and to perform a functional enrichment analysis specific for Biological Process (GO) and KEGG Pathways, using the STRING database. Line thickness on the interaction network indicates the strength of data support. Proteins were clustered and enriched functions are indicated. The expression of (**c**) IRF4, (**d**) Hsp90 and (**e**) CD3e was assessed by flow cytometry to validate proteins identified through the proteomic analysis. Data from three independent individuals are shown as mean ± SEM. Significance of pairwise comparisons between groups is indicated with stars (*p < 0.05, **p < 0.01, ***p < 0.001)

To gain a more in-depth view of the role that K_v_1.3 channels play in T_EM_ cell activation, we performed a gel-free and label-free mass spectrometry-based quantitative proteomic analysis. As for previous experiments, CD4^+^ T_EM_ cells from three independent donors were left unstimulated or were activated for 24 h with plate-bound OKT3, both in the presence or absence of the Vm24 toxin. The proteomic analysis yielded 1013 different proteins across the dataset (Additional file 2). Comparison between the four experimental groups using one-way ANOVA (*p* < 0.05) as a first threshold, uncovered 90 proteins differentially expressed across the dataset. The comparison of the unstimulated and the OKT3-stimulated group detected those proteins with at least 1.5-fold change in either direction that were significantly (*p* < 0.05) different, and are indicated in the upper right and left segments of the volcano plot (Fig. 4a). Out of the 53 proteins modified upon TCR engagement, 38 were upregulated and 15 were downregulated (Table 1), a list consistent (81.1% agreement) with previously published proteomic data from TCR activated T cells [38, 39]. Within the OKT3-regulated proteins, a pairwise comparison between the OKT3 and the OKT3 + Vm24-treated group identified 27 proteins (red spots in the volcano plot, *p* < 0.05) affected by K_v_1.3 channel blockade in a T cell activation context. Overall, these 27 proteins preserved the same trend of change than when cells where stimulated with OKT3 only. However, the amplitude of the change was reduced, evidencing an inhibition of the TCR-mediated signals as a result of K_v_1.3 blockade (Fig. 4a and Table 1).

**Table 1:** Proteins identified in the quantitative proteomic analysis differentially expressed across the dataset

UniProt KB	Gene	Protein	Fisher’s LSD (*p*) US vs. OKT3	Fisher’s LSD (*p*) OKT3 vs. OKT3 + Vm24	Fold change OKT3/US	Fold change OKT3 + Vm24/US	Concordance with other proteomic studies^a^
Q15306	***IRF4***	Interferon regulatory factor 4	1.4E-06	1.53E-04	13.04	6.71	1, 2
Q13283	***G3BP1***	Ras GTPase-activating protein-binding protein 1	7.5E-06	1.72E-05	3.90	1.31	1, 2
Q9Y3F4	***STRAP***	Serine-threonine kinase receptor-associated protein	8.98E-06	8.98E-06	13.95	1.00	1, 2
P14625	***HSP90B1***	Endoplasmin	5.94E-05	2.39E-04	2.03	1.19	1, 2
P08238	***HSP90AB1***	Heat shock protein HSP 90-beta	2.02E-04	1.26E-03	1.94	1.23	1, 2
P98179	***RBM3***	RNA-binding protein 3	1.49E-04	3.11E-04	2.43	1.15	1, 2
P35606	***COPB2***	Coatomer subunit beta’	8.85E-04	1.20E-03	1.90	1.04	1, 2
Q92598	***HSPH1***	Heat shock protein 105 kDa	5.92E-04	2.44E-02	4.26	2.61	1, 2
P53618	***COPB1***	Coatomer subunit beta	5.90E-04	1.05E-02	3.67	2.05	1, 2
P11142	***HSPA8***	Heat shock cognate 71 kDa protein	8.09E-04	4.05E-03	1.87	1.20	1, 2
P31153	***MAT2A***	S-adenosylmethionine synthase isoform type-2	1.47E-03	5.27E-03	7.91	2.37	1, 2
Q2YHR9	***HLA-B***	HLA class I histocompatibility antigen	2.08E-03	4.51E-03	2.23	1.16	1
P62995	***TRA2B***	Transformer-2 protein homolog beta	1.43E-03	7.12E-03	3.43	1.60	2
P11021	***HSPA5***	78 kDa glucose-regulated protein	3.24E-03	1.32E-02	1.62	1.14	1, 2
O43396	***TXNL1***	Thioredoxin-like protein 1	1.26E-03	3.45E-02	4.61	2.72	1, 2
Q14103	***HNRNPD***	Heterogeneous nuclear ribonucleoprotein D0	5.12E-03	1.60E-02	1.67	1.14	2
Q13435	***SF3B2***	Splicing factor 3B subunit 2	4.02E-03	3.16E-02	3.30	1.80	1, 2
Q15436	***SEC23A***	Protein transport protein Sec23A	5.22E-03	2.28E-02	3.64	1.69	2
Q07666	***KHDRBS1***	KH domain-containing, RNA-binding, signal transduction-associated protein 1	1E-02	8.73E-03	2.36	0.96	2
Q16629	***SRSF7***	Serine/arginine-rich splicing factor 7	1.28E-02	1.56E-02	2.99	1.08	2
P08195	***SLC3A2***	4F2 cell-surface antigen heavy chain	9.01E-05	1.88E-02	9.94	6.31	1, 2
O94925	***GLS***	Glutaminase kidney isoform, mitochondrial	1.59E-04	4.37E-02	6.80	4.72	2
P23381	***WARS***	Tryptophan--tRNA ligase, cytoplasmic	8.15E-04	4.82E-02	5.32	3.39	1, 2
O60763	*USO1*	General vesicular transport factor p115	1.07E-03	1.31E-01	3.15	2.42	2
P42224	*STAT1*	Signal transducer and activator of transcription 1-alpha/beta	2.48E-03	1.26E-01	2.98	2.20	2
P10515	*DLAT*	Dihydrolipoyllysine-residue acetyltransferase component of pyruvate dehydrogenase complex, mitochondrial	1.75E-03	9.08E-02	5.30	3.50	2
P23246	*SFPQ*	Splicing factor, proline- and glutamine-rich	3.63E-03	3.23E-01	1.59	1.44	2
O00571	*DDX3X*	ATP-dependent RNA helicase DDX3X	1.07E-02	2.31E-01	3.43	2.48	1, 2
P24534	*EEF1B2*	Elongation factor 1-beta	4.62E-02	1.9E-01	1.54	1.88	1, 2
P17980	*PSMC3*	26S proteasome regulatory subunit 6A	3.69E-02	9.95E-01	1.74	1.73	1, 2
P27708	*CAD*	CAD protein	6.06E-03	1.14E-01	1.93	1.48	1, 2
P17987	*TCP1*	T-complex protein 1 subunit alpha	1.98E-02	3.51E-01	2.58	2.04	1, 2
Q99613	*EIF3C*	Eukaryotic translation initiation factor 3 subunit C	3.55E-03	5.77E-02	3.33	2.06	1, 2
Q9UN86	*G3BP2*	Ras GTPase-activating protein-binding protein 2	9.56E-03	1.17E-01	3.20	2.06	1, 2
P67809	*YBX1*	Nuclease-sensitive element-binding protein 1	1.61E-02	7.19E-02	2.02	1.32	1, 2
P26641	*EEF1G*	Elongation factor 1-gamma	2.7E-02	8E-02	1.76	1.20	1, 2
P34932	*HSPA4*	Heat shock 70 kDa protein 4	1.93E-02	9.42E-02	2.17	1.41	1, 2
P11940	*PABPC1*	Polyadenylate-binding protein 1	1.15E-05	7.50E-02	2.92	2.51	1, 2
P61088	***UBE2N***	Ubiquitin-conjugating enzyme E2 N	4.75E-02	1.82E-03	0.50	1.48	–
P61247	***RPS3A***	40S ribosomal protein S3a	1.48E-03	1.65E-02	0.42	0.79	–
Q53EL6	***PDCD4***	Programmed cell death protein 4	1.26E-03	2.79E-02	0.26	0.67	1, 2
O00160	***MYO1F***	Unconventional myosin-If	6.13E-03	3.84E-02	0.52	0.84	–
Q5JSL3	*DOCK11*	Dedicator of cytokinesis protein 11	1.63E-03	8.98E-01	0.45	0.44	1
Q92522	*H1FX*	Histone H1x	1.97E-03	6.06E-02	0.46	0.72	–
P07737	*PFN1*	Profilin-1	3.51E-03	5.64E-01	0.47	0.55	1
O43390	*HNRNPR*	Heterogeneous nuclear ribonucleoprotein R	4.43E-03	1.9E-01	0.65	0.78	–
Q13561	*DCTN2*	Dynactin subunit 2	1.12E-02	2.14E-01	0.27	0.57	–
P16104	*H2AFX*	Histone H2AX	4.24E-02	9.79E-02	0.52	0.89	–
O94906	*PRPF6*	Pre-mRNA-processing factor 6	2.05E-02	8.37E-02	0.41	0.82	–
P62906	*RPL10A*	60S ribosomal protein L10a	9.61E-03	7.94E-02	0.26	0.70	–
P07766	*CD3E*	T-cell surface glycoprotein CD3 epsilon chain	2.02E-06	1	0.03	0.03	1
Q9UGI8	*TES*	Testin	9.03E-03	4.25E-01	0.66	0.74	–
Q6JBY9	*RCSD1*	CapZ-interacting protein	1.86E-02	2.26E-01	0.20	0.56	1

Proteins with at least 1.5-fold change in either direction that were significantly (*p* < 0.05) different comparing the unstimulated and the OKT3-stimulated group. Concordance with other T cell activation proteomic studies is indicated. Within the OKT3-regulated proteins, comparison between the OKT3 and the OKT3 + Vm24-treated group is indicated and proteins that showed a statistically significant reduction on the amplitude of the TCR-mediated change as a result of the addition of the Vm24 toxin are indicated in bold

a) Concordance with other proteomic studies:

^1^Tan H, Yang K, Li Y, Shaw TI, Wang Y, Blanco DB, et al. Integrative Proteomics and Phosphoproteomics Profiling Reveals Dynamic Signaling Networks and Bioenergetics Pathways Underlying T Cell Activation. Immunity. 2017;46:488–503

^2^Ron-Harel N, Santos D, Ghergurovich JM, Sage PT, Reddy A, Lovitch SB, et al. Mitochondrial Biogenesis and Proteome Remodeling Promote One-Carbon Metabolism for T Cell Activation. Cell Metab. 2016;24:104–17

To gain a wider view of the cellular functions affected by blocking the K_v_1.3 channels in a T cell activation context, the 27 proteins were combined with the activation markers and the cytokine data set, to generate a protein-protein interaction network and to perform a functional enrichment analysis with the STRING database. The biological processes most affected by blocking the K_v_1.3 channel with the Vm24 toxin in a T cell activation context were cytokine-cytokine receptor interaction, mRNA processing, response to unfolded proteins and intracellular vesicle transport (Fig. 4b).

All proteins in the connected clusters were upregulated upon TCR engagement, and the addition of Vm24 partially prevented their upregulation. This data highlights the role of K_v_1.3 channels in reinforcing the TCR-mediated signals, and unravels regulatory functions for these channels in the protein synthesis machinery of T_EM_ cells, further compromising their effector functions at different levels.

Only the TCR-mediated downregulation of four proteins (*RPS3A*, *PDCD4*, *UBE2N* and *MYO1F*) was prevented by the addition of Vm24 (Fig. 4a upper left segment of the volcano plot and Table 1). These proteins are not part of any of the indicated functional clusters.

The comparison of the unstimulated and the Vm24-only treated group, using the same analytical criteria (at least 1.5-fold change in either direction and *p* < 0.05), showed that incubating the unstimulated cells with the Vm24 toxin resulted in the upregulation of three proteins and the downregulation of 11 proteins (Additional file 3). Although these proteins are not involved together in a clear canonical biological process according to the enrichment analysis, they participate in transcriptional activation, DNA repair, RNA stability, ribosomal function, and cytokinesis, suggesting that, in resting T_EM_ lymphocytes, K_v_1.3-mediated signaling also controls essential biological processes.

To validate the proteomic data set, we analyzed the expression level of interferon regulatory factor 4 (*IRF4*) and heat shock protein 90 (Hsp90), as their expression augmented in a TCR-dependent activation context, and when T cells were activated with OKT3 in the presence of the Vm24 toxin this increase was reduced. IRF4, a transcription factor linking the “cytokine-cytokine receptor interaction” cluster with the “intracellular vesicle transport” and “response to unfolded proteins” clusters, is expressed in all CD4^+^ T cell subsets following TCR engagement and it is crucial for cytokine production by effector cells [40]. Hsp90, a fundamental member of the “response to unfolded protein” cluster, participates in the correct folding of the numerous nascent proteins produced during the T lymphocyte effector response and in the regulation of NF-κB signaling and inflammatory responses [41]. Both, IRF4 (Fig. 4c and Additional file 4a) and Hsp90 (Fig. 4d and Additional file 4b) followed the same expression pattern by mass spectrometry and flow cytometry: a marked upregulation following TCR ligation and a partial reduction of this increase in the presence of either Vm24 or ShK toxins, indicating that K_v_1.3 channel function is important for their upregulation. We also evaluated CD3e (*CD3E*) since TCR stimulation either by antigen, anti-CD3 antibodies, or pharmacological activators of protein kinase C, results in increased TCR-CD3 internalization and therefore, a down-modulation of its surface levels [42, 43]. Under our experimental conditions, the OKT3-dependent T_EM_ cell activation resulted in an almost complete disappearance of CD3e from the T_EM_ cells surface, regardless of the presence of Vm24 or ShK, suggesting that the molecular events regulating CD3e down-modulation are not dependent on the activity of the K_v_1.3 channels (Fig. 4e and Additional file 4c).

Altogether, our results identified a number of proteins targeted by the K_v_1.3 channel-dependent signaling in TCR-activated CD4^+^ T_EM_ lymphocytes, uncovering regulatory roles for K_v_1.3 channels in mRNA processing, response to unfolded proteins, intracellular vesicle transport and cytokine-cytokine receptor interaction.

## Discussion

The potassium channels K_v_1.3 and K_Ca_3.1 promote the sustained Ca^2+^ influx necessary for complete T cell activation. Particularly, K_v_1.3 channels are highly expressed in T_EM_ cells and regulate their activity [6]. Inhibition of K_v_1.3 channels by pharmacological blockers has been shown to inhibit the Ca^2+^-dependent response to antigen stimulation and to ameliorate autoimmune diseases such as multiple sclerosis and psoriasis in animal models [13, 44]. In the present study, we used the Vm24 toxin, a highly specific blocker of the K_v_1.3 channels to identify the cellular processes that depend on the activity of these channels in TCR-activated human T_EM_ lymphocytes as well as to validate the use of the Vm24 peptide to downregulate T_EM_ cell function.

Consistent with previous reports [23], and with the high dependency of T_EM_ lymphocytes on K_v_1.3 channel function for sustained activation, the addition of Vm24 to CD3-activated T_EM_ lymphocytes resulted in a pronounced inhibition of CD25 and CD40L expression, probably as a consequence of the lack of activation of calcineurin and the subsequent translocation of the NFAT transcription factor to the CD25 and CD40L promoters [45] [46]. Interestingly, under our experimental conditions, Vm24 did not prevent the OKT3-induced expression of CD69, a negative regulator of chronic inflammation [47, 48].

In consonance with the inhibition of the expression of early activation markers, the addition of the Vm24 peptide to OKT3-activated T_EM_ lymphocytes inhibited the secretion of the pro-inflammatory cytokines IFN-γ and TNF, as well as that of the Th2 cytokines IL-4, IL-5, IL-9, IL-10 and IL-13, all of which are dependent on the availability of NFAT [49–59].

We previously reported that administration of the Vm24 peptide lessened the severity of inflammation in a delayed-type hypersensitivity model [23]. Other studies in animal models of human diseases (allergic asthma, experimental autoimmune encephalomyelitis) have shown that the abatement of the K_v_1.3 channel currents in vivo (by knocking out the channel or by treatment with ShK derivatives) ameliorates the progression of the disease and decreases the production of the effector cytokines IFN-γ, IL-4, IL-5 and IL-17, but enhances that of the anti-inflammatory cytokine IL-10 [60, 61]. Contrary to these reports, under our experimental conditions, the blockade of K_v_1.3 channels in isolated human CD4^+^ T_EM_ cells from healthy donors reduced IL-10 production, consistent with a downstream inhibition of NFATc2 and IRF4 recruitment, two transcription factors that synergistically augment the activity of the Th2-specific enhancer CNS-9 (a cis-regulatory element upstream of the IL-10 gene locus) [62, 63]. Whether this discrepancy reflects species-specific differences or differences that result from different experimental settings (knocking out the channel or an in vivo T cell activation versus in vitro anti-CD3 CD4^+^ T_EM_ activation) remains to be investigated.

Also, it is important to consider that although IL-4, IL-5, IL-10 and IL-13 are classified as anti-inflammatory cytokines due to their down-modulatory effect on inflammatory phenomena mediated by Th1/Th17 cells, these cytokines mediate type I hypersensitivity inflammatory conditions and significantly contribute to the pathogenesis mediated by immune complexes, through their important effect on the activation and proliferation of B cells and antibody synthesis [64].

Furthermore, consistent with the fact that the signaling pathways that activate the transcription of those genes are also dependent on NFAT availability, our data suggest that the inhibition of the K_v_1.3 channel exerts a down-regulatory effect on the different T_EM_ lymphocyte subsets, including Th1 and Th2 cells. In this regard, it would be expected that K_v_1.3 blockers could act as a wide spectrum immunosuppressive molecule, with a significant effect on different immune-mediated conditions.

The proteomic analysis revealed that, in agreement with the large secretory demand of TCR-activated T_EM_ cells, the protein synthesis machinery is prepared to generate a robust immune response by regulating the expression level of transcription factors specific for inflammatory mediators, such as IRF4, as well as that of proteins involved in the splicing machinery, the unfolded protein response and vesicular transport of the novel synthesized mediators. IRF4 is expressed across all T cell subsets within a few hours following TCR engagement, and it is necessary for optimal T cell proliferation in response to mitogenic stimuli. In cooperation with transcriptional partners such as NFAT, it controls the expression of IL-2, IL-4, IL-5, IL-9, IL-10, IL-13, IL-17, IL-21, IFN-γ and TNF [40, 65–67]. Furthermore, the calcineurin inhibitor cyclosporine A [65] as well as defects in CRAC channels function [68] result in impaired IRF4 expression, implicating NFAT in IRF4 upregulation. Our data show that in a T cell activation context, the expression level of IRF4 is diminished following Vm24 treatment, suggesting that blocking the K_v_1.3 channel hinders downstream events such as NFAT activation and IRF4 expression, resulting in impaired cytokine production, in consonance with a previously assigned function for K_v_1.3-dependent signals in secretory functions [14, 69].

The unfolded protein response allows cells to manage the endoplasmic reticulum stress resulting of the increased folding demand imposed by the requirements of activated T lymphocytes engaged in secretory functions. Especially, the molecular chaperone Hsp90 regulates the stability and function of IRE1, an endoplasmic reticulum transmembrane kinase that activates the unfolded protein response to maintain the endoplasmic reticulum function [70]. Upon treatment with the Vm24 peptide or the ShK toxin, the expression level of the Hsp90 chaperone as well as that of other members of the heat shock family of proteins was strongly diminished. In addition, we found that K_v_1.3 channels-dependent signals are necessary for the up-regulation of the amino acid transporter *SLC3A2*. Nutrient transporters such as *SLC3A2* ensure and coordinate the supply of nutrients necessary for the increased metabolic requirements of effector lymphocytes. Thus, K_v_1.3 channels participate in regulating basic metabolic requirements in T_EM_ cells. In concordance with our results, a recent proteomic study performed on activated microglia also revealed that blocking K_v_1.3 channels attenuates biological processes related to the regulation of the immune response and the intracellular protein transport [71]. Assessing if this extends to other cell types becomes critical to appreciate better the potential side effects of blocking the K_v_1.3 channels to control autoimmune disorders.

Interestingly, we found that blocking the K_v_1.3 channels with Vm24 on OKT3-activated CD4+ T_EM_ cells does not entirely block the anti-CD3 induced cytokines synthesis or proteomic changes; it prevents the changes from reaching the highest level. Residual potassium fluxes from the few K_Ca_3.1 channels present on these cells may contribute to the “incomplete” inhibition [35]. In support of the relative abundance of K_v_1.3 channels as compared to that of K_Ca_3.1 channels on T_EM_ cells, our data indicate that K_v_1.3 channels are the major providers of potassium efflux; they are not indispensable for CD4^+^ T_EM_ cell response, yet they are central for regulating the amplitude of the response.

Although less is known about the function of the K_v_1.3 channels in human CD8^+^ cells, the decrease of K_v_1.3 currents by genetic or pharmacological approaches inhibits the differentiation of T_CM_ to T_EM_ cells and in the latter, it severely impairs the proliferation, the secretion of IL-2, TNF and granzyme B (but not of IFN-γ), ultimately dampening their ability to kill target cells [69, 72]. Interestingly, NFATc1-deficient CD8^+^ T cells show diminished RNA levels of granzyme B and of genes encoding cytokines and chemokines in addition to genes controlling glycolysis [73], further underscoring the importance of K_v_1.3 channels on the calcium/calcineurin/NFAT network.

In addition to exploring the function of K_v_1.3 channels in activated CD4^+^ T_EM_ cells, the experiments we performed allowed us to compare the efficacy of two K_v_1.3 channel blockers: the Vm24 toxin, isolated from the Mexican scorpion *Vaejovis mexicanus* and the ShK toxin, from the sea anemone *Stichodactyla helianthus*. Their capacity to hinder T_EM_ cells functions showed that both toxins were very effective to block the K_v_1.3 potassium channels-dependent signaling as they both inhibited the production of INF-γ, TNF, IL-4, IL-5, IL9, IL10 and IL13 following TCR ligation. Although not statistically significant, the inhibitory capacity of the ShK peptide was always higher than that of Vm24. The ShK peptide is a very potent (K_d_ = 10 pM) K_v_1.3 blocker. However, it has a low selectivity for the K_v_1.3 channels (only 2.8-fold affinity over other channels), as a result of which it is very toxic to mammals [21, 74]. Dalazatide® (formerly ShK-186), a molecule derived from the ShK toxin, is a K_v_1.3 channel blocker that has gone into clinical phase trials, has been shown to reduce the levels of plasmatic and T_EM_ inflammation markers, and to improve psoriatic skin lesions from mild to moderate plaque psoriasis patients [13, 16, 75]. In comparison with Dalazatide®, the Vm24 peptide has the advantage of a greater potency (K_d_ 2.9 vs. 69 pM) and selectivity (1500 vs. 100-fold affinity over other channels) towards K_v_1.3 channels [21, 23], which improves the safety index in conditions where the blood-brain barrier (such as multiple sclerosis) is compromised and the presence of high concentrations of non-specific blockers (such as neuronal K_v_1.1 channels blockers) in the central nervous system can bring severe neurotoxicity. Interestingly, although Dalazatide® was well tolerated during clinical trials, the most common adverse events were temporary hypoesthesia and paresthesia involving hands, feet, or perioral area which may be prevented with more selective K_v_1.3 blockers such as Vm24 or other second-generation blockers derived from ShK toxin that have been recently developed [76].

## Conclusions

Our results show that blocking K_v_1.3 channels with the Vm24 peptide profoundly affects the mRNA synthesis machinery, the unfolded protein response and the intracellular vesicle transport, thus impairing the synthesis and secretion of cytokines in response to TCR engagement, highlighting the importance of K_v_1.3 channels for T_EM_ cell function. As T_EM_ cells are considered to be main players in the pathology of autoimmune diseases, further studies are needed to better characterize the molecular mechanisms affected by the blockade of K_v_1.3 channels by toxins such as the Vm24 peptide.

## Additional files

Additional file 1: K_v_1.3 channel blockade does not compromise cell viability. (**a**) Cell viability of cells treated under the same experimental conditions as for Fig. 1 was assessed following a 24, 48 and 96 h culture period with the Fixable Viability Dye eFluor 780. The dot plot of a representative donor after 24 h of culture is shown. Changes in forward scatter (FSC) and positive staining with the viability dye were considered as cell death markers. As death positive control, 30% dimethyl sulfoxide (DMSO) was added to the cells for 30 min. The cell death area is enclosed in the gate. (**b**) Data from three independent experiments with CD4^+^ T_EM_ cells from independent donors are shown as mean ± SEM. (TIF 6948 kb)

Additional file 2: Proteins identified in the mass spectrometry-based quantitative proteomic analysis. Peptide identifications were accepted if they could be established at greater than 95% probability as specified by the PeptideProphet algorithm. Protein identifications were accepted if they could be established at greater than 99% probability. (XLSX 427 kb)

Additional file 3: Proteins differentially expressed when incubating the unstimulated cells with the Vm24 toxin. Proteins identified with the mass spectrometry-based quantitative proteomic analysis, with at least 1.5-fold change in either direction and that were significantly (*p* < 0.05) different, comparing the unstimulated and the Vm24-treated group. (PDF 150 kb)

Additional file 4: Validation of proteomic analysis results by flow cytometry. CD4+ T_EM_ cells were stimulated through the TCR with plate-bound OKT3 in the presence or absence of Vm24 or ShK (1 nM) toxins, as indicated in the methods section. After 24 h of culture, cells were stained for (**a**) IRF4, (**b**) Hsp90 and (**c**) CD3e. The histogram of one representative donor for each staining is shown. (TIF 4252 kb)

## Abbreviations

NF-κB: nuclear factor kappa-light-chain-enhancer of activated B cells
CRAC: Ca^2+^ release-activated Ca^2+^ channels
DMSO: Dimethyl sulfoxide
emPAI: Exponentially modified protein abundance index
Fisher’s LSD: Fisher’s Least Significant Difference post-hoc test
FSC: Forward scatter
Hsp90: Heat shock protein 90
IRE1: Serine/threonine-protein kinase/endoribonuclease IRE1
IRF4: Interferon regulatory factor 4
K_d_: Dissociation constant
MS/MS: Tandem mass spectrometry
NFAT: Nuclear factor of activated T-cells
SEM: Standard error of mean
TCR: T cell receptor
T_EM_: Effector memory T cells
