# Supplementary figures and images for: Kv1.3 channel blockade with the Vm24 scorpion toxin attenuates the CD4+ effector memory T cell response to TCR stimulation
Source: Cell Commun Signal. 2018 Aug 14;16:45. doi: 10.1186/s12964-018-0257-7 (PMC6092819; doi:10.1186/s12964-018-0257-7)

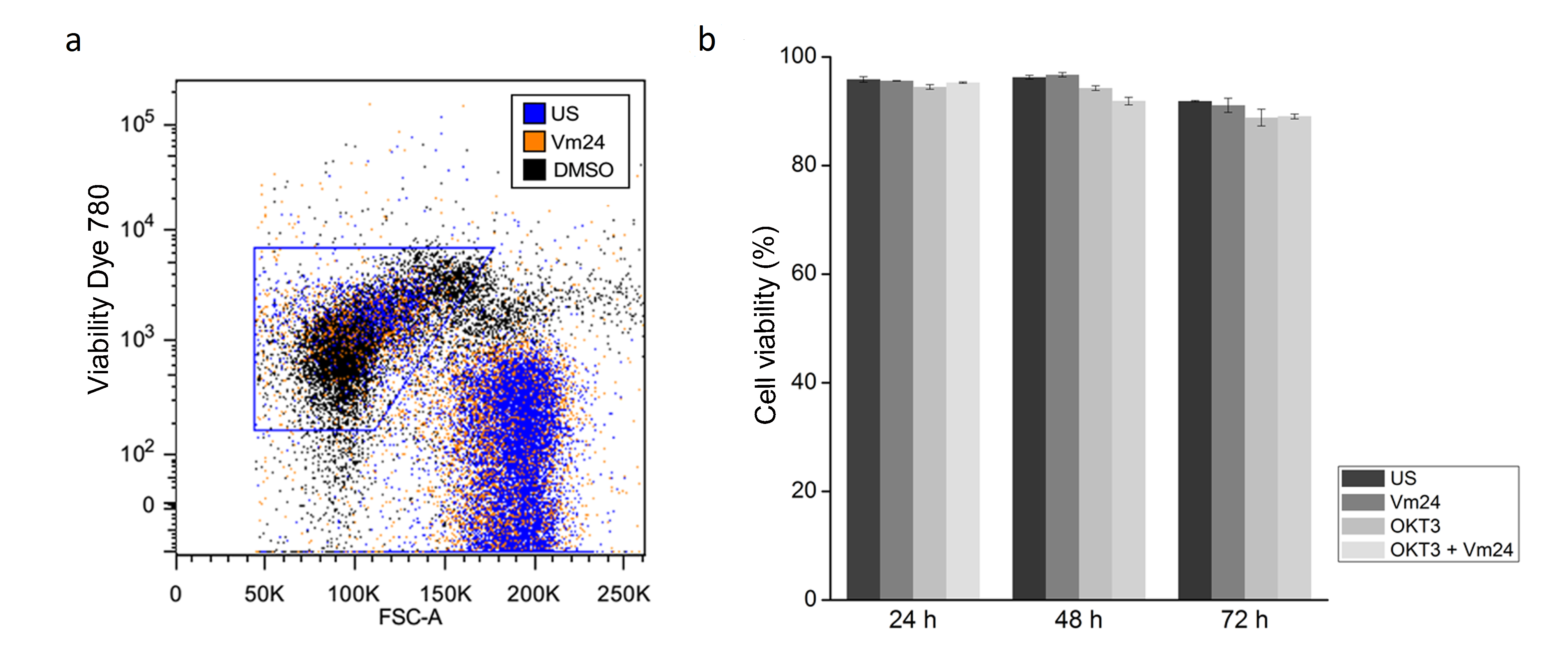

Supplement: Supplementary file 1 — Kv1.3 channel blockade does not compromise cell viability. (a) Cell viability of cells treated under the same experimental conditions as for Fig. 1 was assessed following a 24, 48 and 96 h culture period with the Fixable Viability Dye eFluor 780. The dot plot of a representative donor after 24 h of culture is shown. Changes in forward scatter (FSC) and positive staining with the viability dye were considered as cell death markers. As death positive control, 30% dimethyl sulfoxide (DMSO) was added to the cells for 30 min. The cell death area is enclosed in the gate. (b) Data from three independent experiments with CD4+ TEM cells from independent donors are shown as mean ± SEM. (TIF 6948 kb) [file 12964_2018_257_MOESM1_ESM.tif]

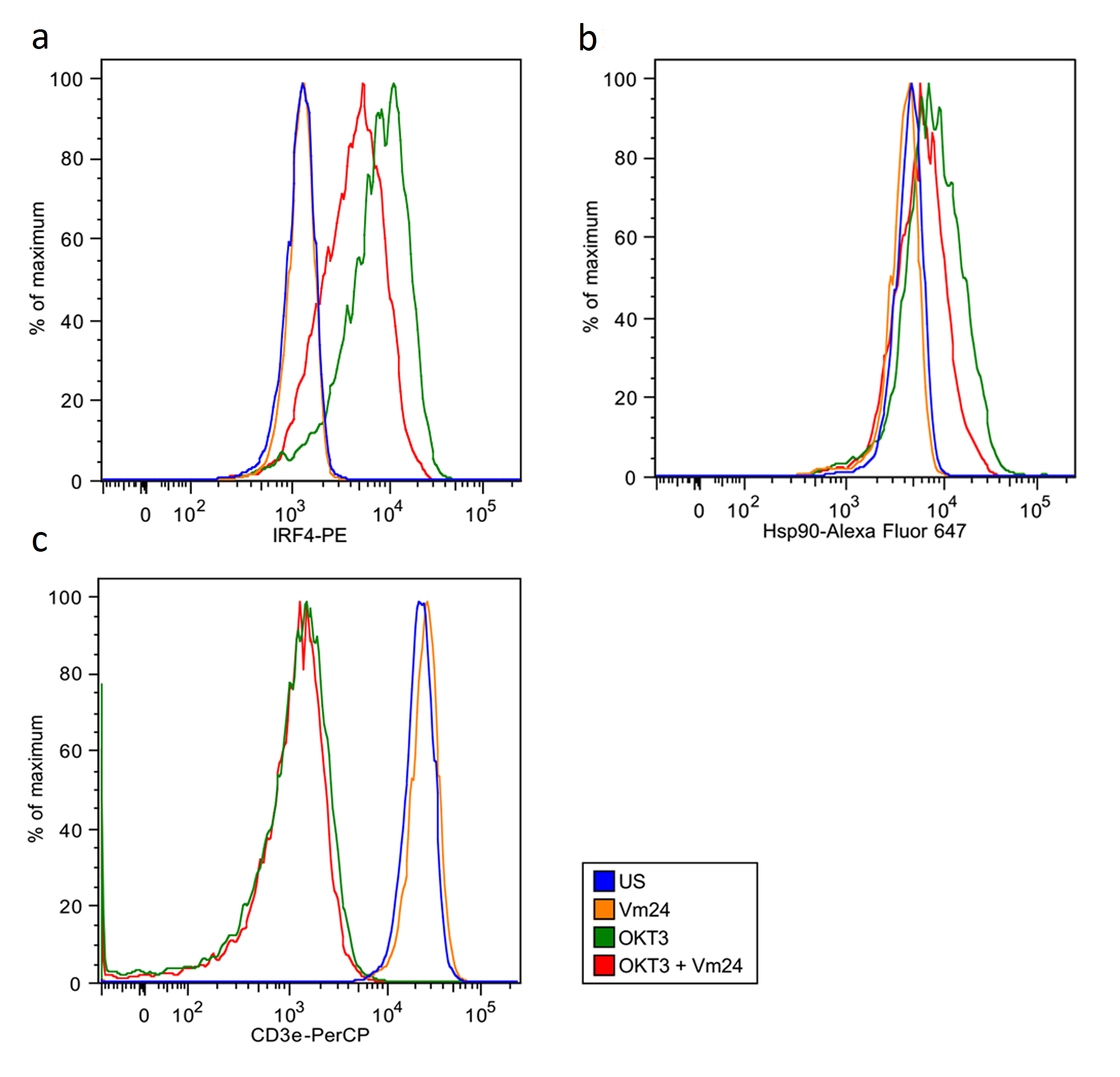

Supplement: Supplementary file 4 — Validation of proteomic analysis results by flow cytometry. CD4+ TEM cells were stimulated through the TCR with plate-bound OKT3 in the presence or absence of Vm24 or ShK (1 nM) toxins, as indicated in the methods section. After 24 h of culture, cells were stained for (a) IRF4, (b) Hsp90 and (c) CD3e. The histogram of one representative donor for each staining is shown. (TIF 4252 kb) [file 12964_2018_257_MOESM4_ESM.tif]
