# Supplementary material for: Kv1.3 channel blockade with the Vm24 scorpion toxin attenuates the CD4+ effector memory T cell response to TCR stimulation
Source: Cell Commun Signal. 2018 Aug 14;16:45. doi: 10.1186/s12964-018-0257-7 (PMC6092819; doi:10.1186/s12964-018-0257-7)
Supplement: Supplementary file 3 — Proteins differentially expressed when incubating the unstimulated cells with the Vm24 toxin. Proteins identified with the mass spectrometry-based quantitative proteomic analysis, with at least 1.5-fold change in either direction and that were significantly (p < 0.05) different, comparing the unstimulated and the Vm24-treated group. (PDF 150 kb) [file 12964_2018_257_MOESM3_ESM.pdf]

UniProtKB	Gene	Protein	Fisher´s LSD (p) US vs. Vm24	Fold change Vm24/US
P61254	*RPL26*	60S ribosomal protein L26	4.8E-02	1.84
P61604	*HSPE1*	10 kDa heat shock protein, mitochondrial	1.6E-03	2.68
P61088	*UBE2N*	Ubiquitin-conjugating enzyme E2 N	1.5E-02	1.66
Q5JSL3	*DOCK11*	Dedicator of cytokinesis protein 11	3.8E-03	0.53
Q92522	*H1FX*	Histone H1x	2.1E-02	0.66
Q9Y3L3	*SH3BP1*	SH3 domain-binding protein 1	7.3E-03	0.50
Q13561	*DCTN2*	Dynactin subunit 2	1.9E-02	0.35
P61247	*RPS3A*	40S ribosomal protein S3a	5.0E-03	0.53
P16104	*H2AFX*	Histone H2AX	1.3E-02	0.37
O94906	*PRPF6*	Pre-mRNA-processing factor 6	1.6E-02	0.38
Q93VT9	*RPL10A*	60S ribosomal protein L10-1	3.5E-02	0.44
Q9NYF8	*BCLAF1*	Bcl-2-associated transcription factor 1	2.1E-03	0.24
P61224	*RAP1B*	Ras-related protein Rap-1b	8.9E-03	0.50
Q13283	*G3BP1*	Ras GTPase-activating protein-binding protein 1	4.0E-02	0.30
